# Three-Dimensional Physical Model-Assisted Planning and Navigation for Laparoscopic Partial Nephrectomy in Patients with Endophytic Renal Tumors

**DOI:** 10.1038/s41598-017-19056-5

**Published:** 2018-01-12

**Authors:** Gang Fan, Jun Li, Mingfeng Li, Mingji Ye, Xiaming Pei, Feiping Li, Shuai Zhu, Han Weiqin, Xiao Zhou, Yu Xie

**Affiliations:** 1 Department of Urology, The Affiliated Cancer Hospital of Xiangya School of Medicine of Central South University, Hunan Cancer Hospital, Changsha, 410013 China; 2 School of Public Health, Xiangnan University, Chenzhou, 423000 China; 3 Department of Radiology, The Affiliated Cancer Hospital of Xiangya School of Medicine of Central South University, Hunan Cancer Hospital, Changsha, 410013 China; 4 Department of Clinical translational research center, The Affiliated Cancer Hospital of Xiangya School of Medicine of Central South University, Hunan Cancer Hospital, Changsha, 410013 China

## Abstract

Resection of completely endophytic renal tumors is a huge challenge for surgeons due to a lack of definite visual clues, especially in the laparoscopic approach. Three-dimensional (3D) kidney models, which can illustrate the clear relationship between renal masses and surrounding health tissues, were considered as reliable tools for understanding renal tumor characteristics in previous studies. We hypothesized that 3D kidney models can be used not only for planning but also for navigating laparoscopic partial nephrectomy (LPN) in patients with completely endophytic renal tumors. In this study, we successfully constructed five cases of 3D kidney models for assisted planning and navigation for LPN in endophytic renal tumors. The renal masses and surrounding normal parenchyma of the patient-specific 3D models were dyed by different colorants for clear illustration. All patients experienced acceptable perioperative outcomes, and no patient suffered serious relative complications. The 3D kidney models were considered as a reliable tool based on clinical outcome and postoperative questionnaire results. This study is the first report of 3D kidney models for patients with completely endophytic tumors. 3D kidney models can aid surgeons in understanding the characteristics of renal tumors and potentially support assisted planning and performance of LPN in endophytic tumor cases.

## Introduction

Resection of completely endophytic renal tumors through laparoscopic partial nephrectomy (LPN) is very challenging for surgeons. No definite visual clues are found on the renal surface for interoperation tumor identification, while an insufficient external view can cause difficulty for predicting mass extension.

Three-dimensional (3D) printing is a new technology that can produce high-fidelity 3D models, which reveal the size, depth, and location of the renal masses, arteriovenous systems, and collection systems, to educate patients and trainees and assist the surgeons in preoperative surgical planning^1–3^. In this study, we produced patient-specific 3D physical models for planning and navigating LNP with complete endophytic renal masses. In this paper, we report our preliminary findings.

## Patients and Methods

### Data collection

Data of five patients with completely endophytic renal tumors, who had undergone 3D kidney models-assistant LNP by a single surgeon at our center (the Affiliated Cancer Hospital of Xiangya Medical College, Central South University), were obtained from a clinical database center. The patients had T1N0M0 renal cancer classified by UICC TNM classification of malignant tumors^4^, according to the patients’ preoperative contrast enhanced computed tomography (CT) images, and the tumors were identified as 3 points of exophytic/endophytic properties of the R.E.N.A.L. nephrometry score^5^. The research was approved by the Hunan Cancer Hospital Medical Review Board and Ethical Board, and an informed consent form was signed by each patient before the manufacture of 3D models.

### Surgical technique

#### 3D printing model construction

**Figure 1:**
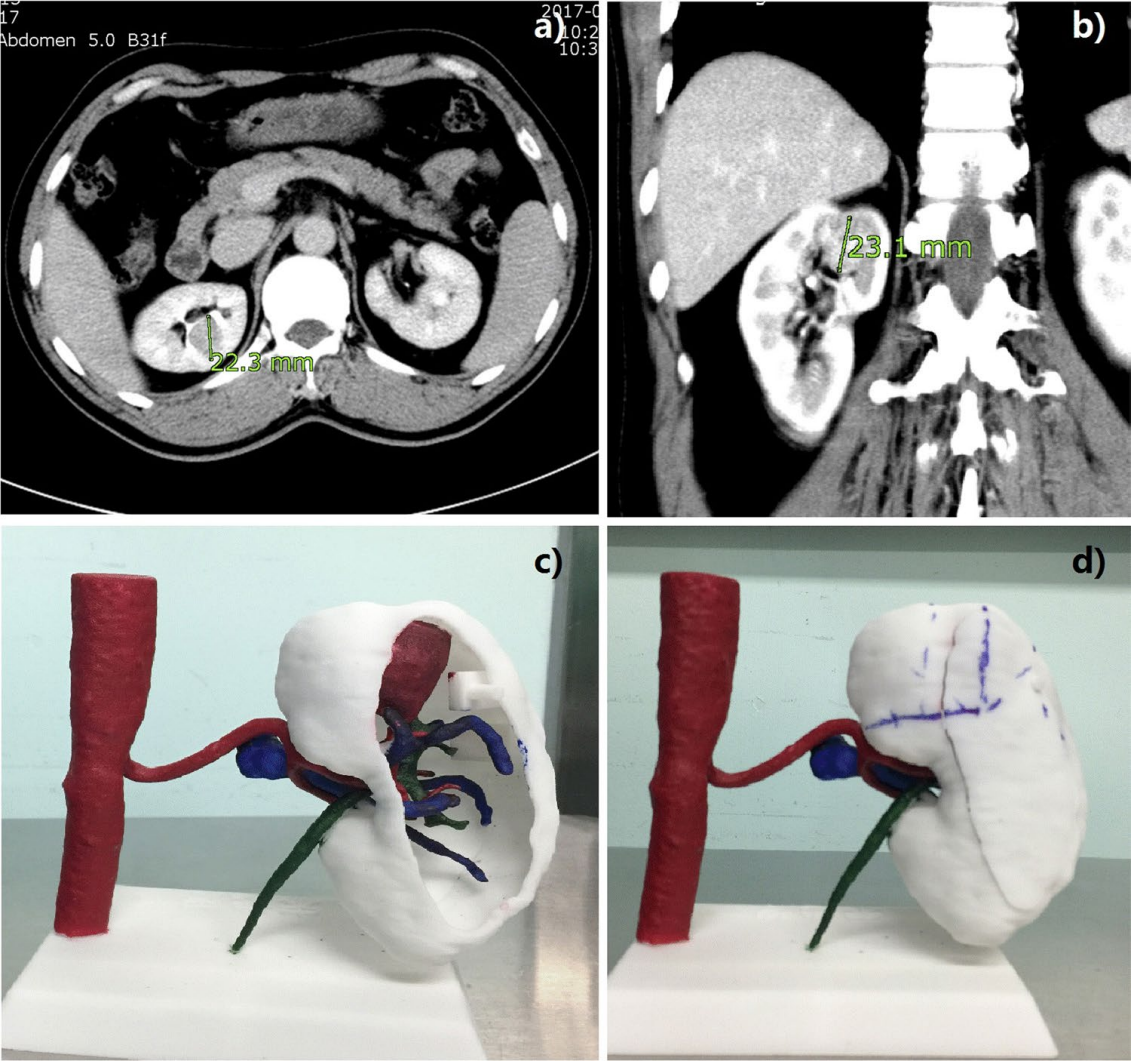
Computed tomography images (**a**,**b**) and 3D kidney model (**c**,**d**). The endophytic mass markers are labeled on the surface of the models after preoperative planning (**d**). The renal artery is an important external anatomical landmark.

Individualized physical 3D anatomic renal models were created as previously described^6,7^. In brief, preoperative contrast enhanced computed tomography (CT) images of the arterial, venous, and pelvis, phases were obtained with 0.75-mm step intervals. We collected the image data and ran them through the Mimics 18.0 system (Materialise, Leuven, Belgium) for 3D reconstruction. FS3200PA Nylon Powder was used to create 3D anatomic models. The printing material was mixed with colorant resin to represent the tumors, arteries, veins, and pelvis in different hues (Fig. 1c) (see Supplementary Fig. S1). The production process was carried out with help from a private company (Huaxiang Incremental Manufacturing Solutions Co., Ltd., Hunan, China).

### 3D kidney models for laparoscopic partial nephrectomy planning

One urologist and one or two radiologists evaluated the reliability of tumor-specific kidney models by comparing them with computed tomography images to reduce deviations. Subsequently, surgeons discussed the complexity and feasibility of LPN and potential complications regarding arteriovenous distribution, collection system, and tumor characteristics.

The markers for endophytic masses were labeled on the surface of the models, which were based on several important anatomical positions of the renal system, such as the hilus and upper and lower renal poles, for mapping the location to guide target dissociation (Fig. 1d), in which the renal artery was the most reliable anatomical landmark. Two renal poles were considered as auxiliary landmarks for assisted renal masses orientation. The technique of hilar clamping and clamping position (renal artery or branch) was also discussed. On the ground of planned occlusion, surgeons determined the retroperitoneal or transperitoneal approach based on 3D renal models for operations.

### 3D kidney models for laparoscopic partial nephrectomy navigating

**Figure 2:**
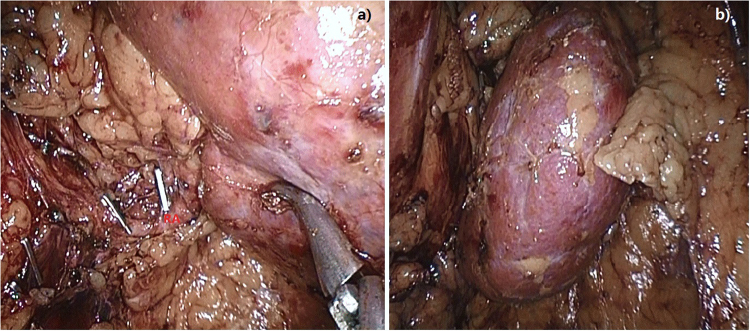
Renal dissociation. The entire renal is fully dissociated from surrounding tissues to expose the renal hilus and the upper/lower renal poles.

3D renal models were used in operative implementation. Here, 3D models were cleaned and disinfected before operation. The entire kidney was fully dissociated from surrounding tissues to expose the renal hilus and the upper/lower renal poles (Fig. 2).

**Figure 3:**
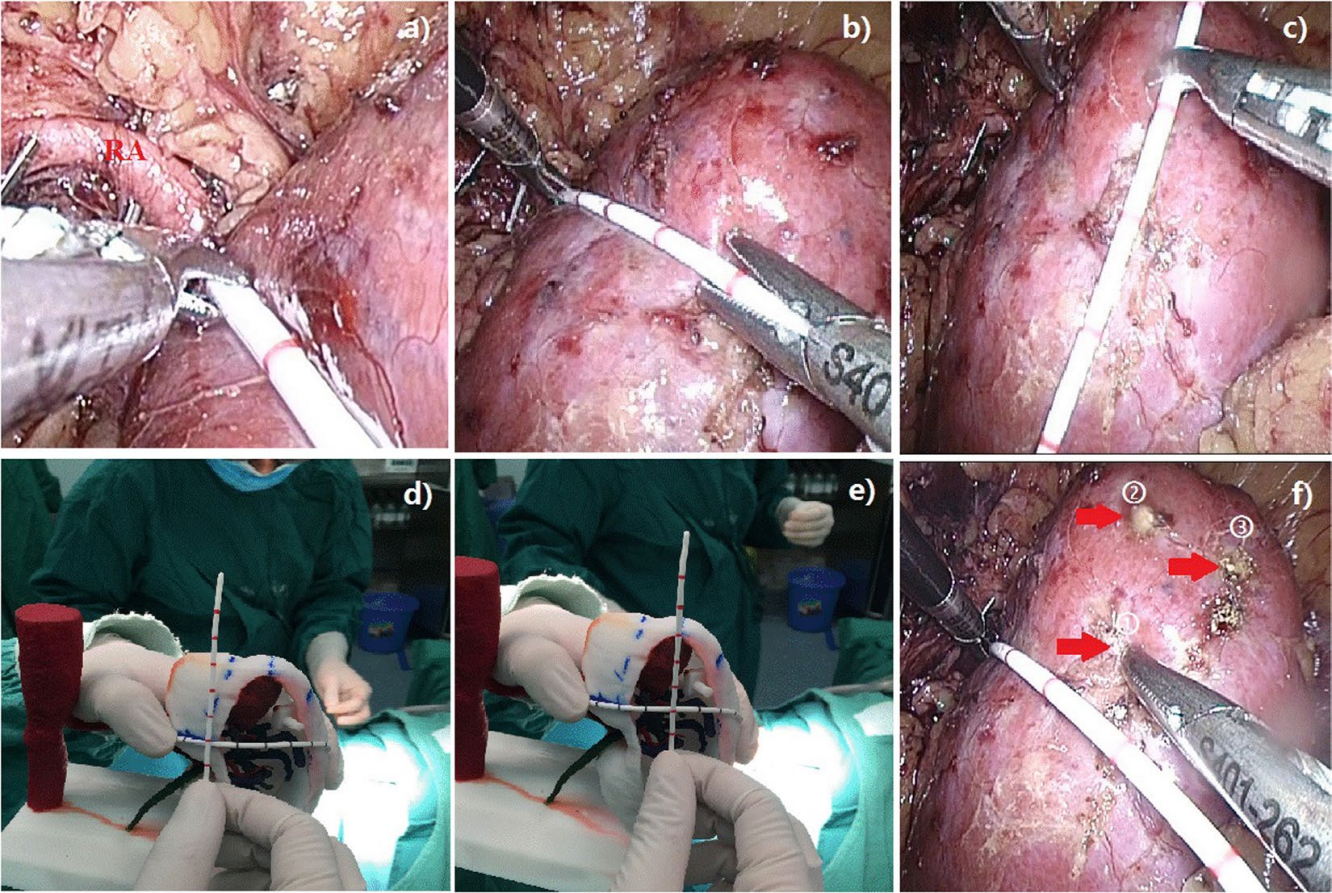
3D physical model in laparoscopic partial nephrectomy. Based on the makers on the surface of the kidney model, a renal mass can be quickly located with precise orientation.

The physical kidney 3D models could map the location of renal masses for guiding target dissociation. A surgical assistant carried the models and rotated them for alignment with the laparoscopic screen image (Fig. 3). This procedure can aid in targeting the operative position based on renal anatomical landmarks and dimensions determined by preoperative planning (Fig. 3). The procedure did not raise interference or increase the burden on the surgeon, but instead aided the surgeon for quick and accurate location of the tumor position and assisted with tumor area exposure (Fig. 3f).

### Questionnaire for model evaluation from surgeon and surgical assistants

A questionnaire consisting of open-ended questions from ordinal 10-point rating scales (1-not at all useful/ not at all realistic/poor, 10-very useful/very realistic/excellent) was plotted from surgical participants^3^. In this questionnaire, 6 items, including overall evaluation (Q1), realism (graphics (Q2a)/details of vasculature and collecting system (Q2b)/tumors size and inter-relationship (Q2c)) and usefulness (for perioperative understanding and planning (Q3a)/for LNP performance (Q3b)), were asked to assess face and content validity.

## Results

Five patients with completely endophytic renal tumors successfully underwent 3D kidney models-assisted LNP performed by the same surgeon with good prevention against damnification of surrounding structures, including the renal arteriovenous and collection system, and without significant intraoperative and postoperative complications. Warm ischemia time was under 30 minutes from 18 minutes to 27 minutes, which met the safety range for renal function protection of under 30 min. At the one-month postoperative visit, the increased creatinine levels of five patients were under 5.4 mg/dl. The pathological examination after operation revealed tumor-free surgical margins. Patient demographics and clinical outcomes are described in Table 1.

**Table 1:** Patient Demographics and Clinical Outcome.

	**Patient 1**	**Patient 2**	**Patient 3**	**Patient 4**	**Patient 5**
**Patient Demographics**					
Age (y)/Sex	58/F	61/M	50/F	53/M	53/M
BMI	24.1	23.7	19.9	23.5	20.8
ASA score	2	1	2	1	2
**Tumor characteristics**					
Tumor histology	RCC	RCC	RCC	RCC	RCC
R.E.N.A.L score (R/E/N/A/L)	1/3/3/2	1/3/3/3	1/3/3/3	1/3/3/3	1/3/3/3
Pathologic stage	T1a	T1a	T1a	T1a	T1a
**Clinical Outcomes**					
Conversion surgical modality	NO	NO	NO	NO	NO
(T)ransperitoneal vs (R)etroperitoneal	R	T	T	T	R
Operative time (minutes)	120	105	199	168	221
Warm ischemia time (minutes)	25	27	24	18	23
Estimated intraoperative blood loss (ml)	50	100	80	50	125
Estimated postoperative blood loss (ml)	200	50	20	20	150
Increased creatinine level (mg/dL)	2.2	5.4	3.0	5.0	3.5
Complications Clavien grade ≥ II	0	0	0	0	0
Tumor margin status	negative	negative	negative	negative	negative
Three-dimensional model deviation (cm)(tumor model size/ tumor size)	0.1(3/2.9)	0.4(3.2/2.8)	0.1(3.6/3.7)	0.1(2.1/2.0)	0.3(2.8/2.5)

^*^ASA, American Society of Anesthesiologists; BMI, body mass index; RCC = renal cell carcinoma.

In this study, the accuracy of 3D models was evaluated and discussed by surgeons and radiologists preoperatively. The postoperative results showed the deviations between the models and renal masses were acceptable, and the average deviation was 0.2 cm.

**Figure 4:**
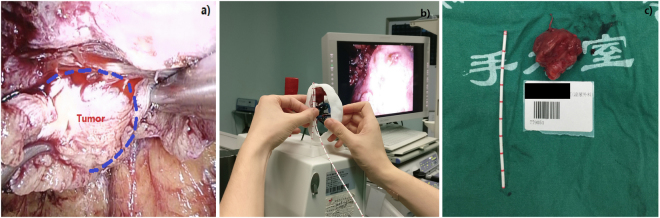
3D kidney model for mass extension prediction.

During surgery, the entire kidney was fully dissociated from surrounding tissues to expose the renal anatomical landmarks. Intraoperative 3D printing models were found to be highly associated with the appearance of the tumor surface. The surgical assistant adjusted the 3D models to aid the surgeon for quickly and accurately locating the site of renal masses based on the dimensions and landmarks on the surface of the 3D kidney models. Thus, endophytic mass resection was successful as planned, and the depth of masses was achieved by the surgeons without any change in surgical modality (Fig. 4). Postoperative review showed the navigation of 3D kidney models potentially help shorten the duration of warm ischemia time for LPN in endophytic tumors.

**Figure 5:**
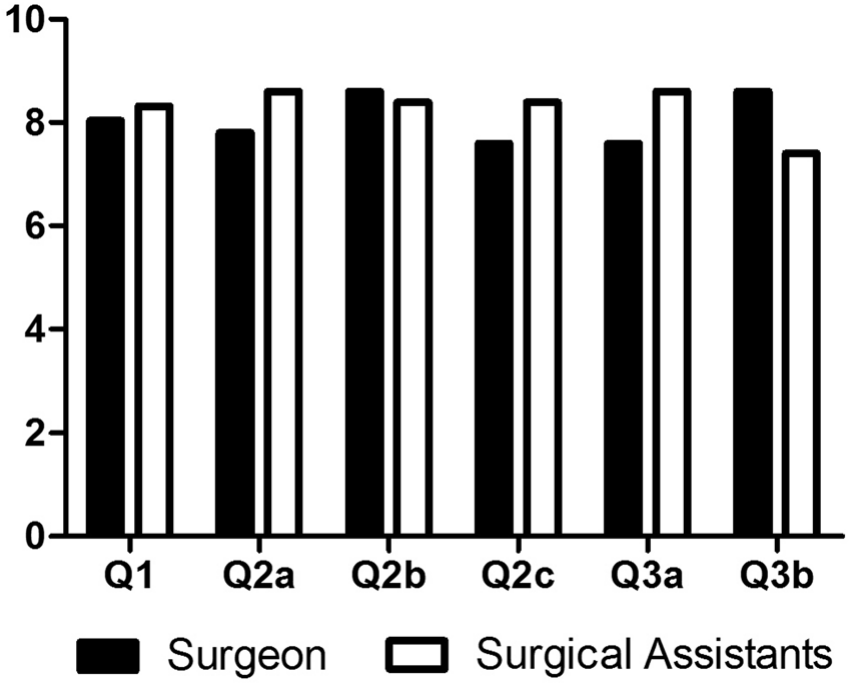
Results to questionnaire regarding 3D physical model for laparoscopic partial nephrectomy in endophytic renal tumors. One operating surgeon and two surgical assistants, were postoperative surveyed to evaluate the helpfulness of 3D model. Q1 overall evaluation; Q2a graphics; Q2b details of vasculature and collecting system; Q2c tumors size and inter-relationship; Q3a perioperative understanding and planning; Q3b intraoperative laparoscopic partial nephrectomy performance.

Postoperatively, three participants, including one operating surgeon and two surgical assistants, were surveyed as part of the assessment for face and content validity. The overall evaluation, realism and usefulness were rated as Fig. 5 showing. All participants advocated that 3D individual kidney models would be a beneficial advancement for helping to improve understanding of endophytic tumors and for performing LPN.

## Discussion

Nephron-sparing surgery is the preferred treatment method for small size and noninvasive renal masses, especially in view of patients with chronic renal disease or/and cardiovascular disease or young age, to attempt to preserve as much normal renal parenchyma as possible. However, minimally invasive LPN for completely endophytic tumors has been hampered by greater technical difficulties, mostly due to the lack of external view.

Robot-assisted partial nephrectomy series have been shown to be feasible and efficient in partial nephrectomy for completely endophytic renal masses^8,9^, with efficiency regarding perioperative complication rates and renal function. Because of the costs of the robot surgical system, the distribution of the robot system is still rare in China. Specifically, the cost per robot operation is two-thirds higher than that of traditional PN.

3D printing has seen rapid advancements in the medical field in recent years because of the creation of individualized high-fidelity 3D models and the decreased cost. The main applications of 3D printing technology in medicine were creating models for surgical planning, practicing, and teaching to create implantable prosthetics, and biologic tissue engineering^10^. Physical kidney models created by 3D printing can provide precise and individualized physical models that help surgeons understand the actual renal tumor characteristics, including tumor size, depth, and the location relationship with the arteriovenous and collection system. Thus, 3D physical models are widely used as a preoperative planning tool that allows surgeons to effectively determine the proper surgical program.

In this study, we hypothesized that 3D printing models not only have an effect on understanding tumor characteristics and perioperative surgical planning but also benefit real-time navigation of LPN in completely endophytic renal tumors, which ultimately shortens warm ischemia time and increases clinical outcome.

Our result showed that the physical construction, which was created by the 3D printing technology based on the computed tomography imaging data of patients, served as an interesting and reliable tool in LNP for patients with completely endophytic tumors. The locations of renal masses models were consistent with the intraoperative findings from our retrospective study. To achieve the position of renal masses quickly and accurately, we performed surgical planning based on patient-specific individualized 3D models. We marked the endophytic mass location on the 3D models surface preoperatively, which is particularly valuable during surgical resection where knowledge of the exact location and depth of the mass is critical. From our perspective, the renal artery appeared to be a reliable landmark for orientation. Two renal poles were considered as auxiliary landmarks for assisted orientation because the polar point in the kidney of some patients is difficult to distinguish laparoscopically. The results from questionnaire showed 3D models useful for both operating surgeon and surgical assistants. The urologists reviewed and claimed that, 3D models can help to predicate the characteristic of endophytic tumors, which is good for preoperative planning, and most importantly, it can also use for individual intraoperative navigation.

Our data demonstrated a safe and feasible approach for surgical planning and implementation in endophytic renal tumors. Warm ischemia time was considered as a crucial factor affecting partial nephrectomy^11^, which was associated with an increased risk of acute renal failure and increased incidence of chronic renal disease. In this study, warm ischemia time for five operations was controlled to be less than 30 minutes, which is currently accepted as the safe range^12^. No apparent postoperative compilations were observed.

The use of ultrasound for recognizing the renal mass position is a common approach in partial nephrectomy, which can provide a rough approximation for the surgery area. However, the huge gap between preoperative planning and intraoperative navigating when there is a lack of preoperative prediction is a huge problem for ultrasound navigating. However, seamless connection between planning and navigation could be achieved in 3D kidney models-assisted surgery. With a tangible copy of the kidney, surgeons may be able to perform good analysis and determinations for surgical navigation. Aside from planning and surgical navigation, 3D models were presented to young surgeons, medical students, and operating staff for training^3^.

3D printing in renal cancer was originally applied for patient and trainee education as well as surgical planning before resection^1–3^. Several studies have illustrated that 3D kidney models could also be used for PN navigation^3,13,14^. 3D models revealed the relationship of masses and surrounding health tissues would be an ideal tool for planning and guiding minimally invasive PN. Jonathan constructed 5 physical models for assisting robot PN or open PN and successfully preserved the surrounding normal renal parenchyma. Komai reported 10 cases of 3D tumor removability models for “4D” surgical navigation in minimally invasive off-clamp partial nephrectomy. The research indicated that 3D kidney models easily allowed surgeons to visualize the optimal outline of the resected tumor. The 3D patient-specific kidney models would be available not only for common masses but also for high complexity masses. Here, we provided an optimal tool for analyzing the anatomy and guiding LNP in endophytic tumors. The proposed 3D kidney model provided a more accurate and predictable result than the traditional model without guided surgery.

## Conclusion

In our research, patient-specific 3D kidney models were utilized as an assisted surgical tool, which help to improve understanding of the renal masses, and their relation to the arteriovenous and collection systems. These models may have the potential to increase anatomic appreciation and improve analysis of the feasibility of nephron sparing to construct a detailed plan and navigation in LPN of patients with completely endophytic tumors.

## Electronic supplementary material

Supplementary Information
