# Supplementary material for: Three-Dimensional Physical Model-Assisted Planning and Navigation for Laparoscopic Partial Nephrectomy in Patients with Endophytic Renal Tumors
Source: Sci Rep. 2018 Jan 12;8:582. doi: 10.1038/s41598-017-19056-5 (PMC5766569; doi:10.1038/s41598-017-19056-5)
Supplement: Supplementary file 1 — Supplementary Information [file 41598_2017_19056_MOESM1_ESM.pdf]

Three-Dimensional Physical Model-Assisted Planning and Navigation for Laparoscopic Partial Nephrectomy in Patients with Endophytic Renal Tumors

Gang Fan[1]●, Jun Li[2]●, Mingfeng Li[1], Mingji Ye[1], Xiaming Pei[1], Feiping Li[3], Shuai Zhu[1], Han Weiqin[1], Xiao Zhou[4], Yu Xie[1]*

Department of [1]Urology, [3]Radiology, [4]Clinical translational research center, the Affiliated Cancer Hospital of Xiangya School of Medicine of Central South University, Hunan Cancer Hospital, Changsha 410013, China.

[2]School of Public Health, Xiangnan University, Chenzhou 423000, China;

** Corresponding author: Yu Xie. [email: 1715840770@qq.com]*

● Both authors contributed equally to this work.

Supplementary Figure S1

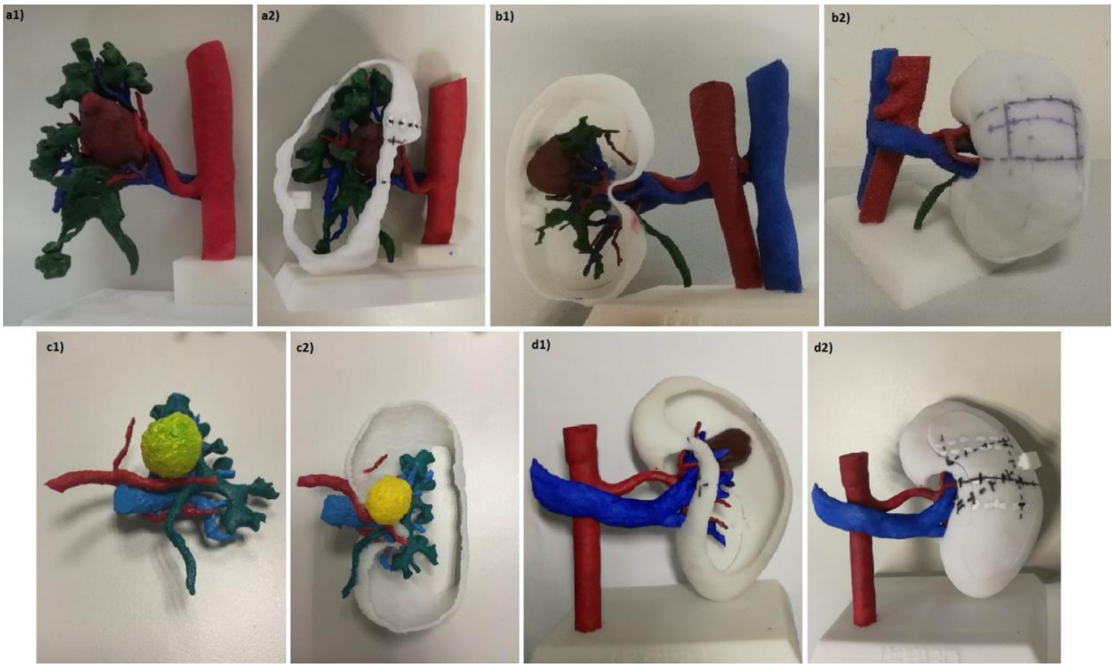

Supplementary Figure Legends

Supplementary Fig. S1. Individualized physical 3D anatomic renal models. Patient 2 (a1, a2), Patient 3 (b1, b2), Patient 4 (c1, c2), Patient 5 (d1, d2).
